# *O*-GlcNAc transferase associates with the MCM2–7 complex and its silencing destabilizes MCM–MCM interactions

**DOI:** 10.1007/s00018-018-2874-0

**Published:** 2018-08-01

**Authors:** Maïté Leturcq, Marlène Mortuaire, Stéphan Hardivillé, Céline Schulz, Tony Lefebvre, Anne-Sophie Vercoutter-Edouart

**Affiliations:** Univ. Lille, CNRS, UMR 8576, UGSF, Unité de Glycobiologie Structurale et Fonctionnelle, 59000 Lille, France

**Keywords:** *O*-GlcNAcylation, Post-translational modification, Pre-replicative complex, DNA replication, Chromatin, Cell cycle

## Abstract

*O*-GlcNAcylation of proteins is governed by *O*-GlcNAc transferase (OGT) and *O*-GlcNAcase (OGA). The homeostasis of *O*-GlcNAc cycling is regulated during cell cycle progression and is essential for proper cellular division. We previously reported the *O*-GlcNAcylation of the minichromosome maintenance proteins MCM2, MCM3, MCM6 and MCM7. These proteins belong to the MCM2–7 complex which is crucial for the initiation of DNA replication through its DNA helicase activity. Here we show that the six subunits of MCM2–7 are *O*-GlcNAcylated and that *O*-GlcNAcylation of MCM proteins mainly occurs in the chromatin-bound fraction of synchronized human cells. Moreover, we identify stable interaction between OGT and several MCM subunits. We also show that down-regulation of OGT decreases the chromatin binding of MCM2, MCM6 and MCM7 without affecting their steady-state level. Finally, OGT silencing or OGA inhibition destabilizes MCM2/6 and MCM4/7 interactions in the chromatin-enriched fraction. In conclusion, OGT is a new partner of the MCM2–7 complex and *O*-GlcNAcylation homeostasis might regulate MCM2–7 complex by regulating the chromatin loading of MCM6 and MCM7 and stabilizing MCM/MCM interactions.

**Electronic supplementary material:**

The online version of this article (10.1007/s00018-018-2874-0) contains supplementary material, which is available to authorized users.

## Introduction

*O*-GlcNAcylation (O-linked β-N-acetylglucosaminylation) of proteins is catalysed by *O*-GlcNAc transferase (OGT) which uses uridine-diphospho-*N*-acetylglucosamine (UDP-GlcNAc) to transfer the GlcNAc moiety onto serine or threonine residues of cytosolic, nuclear and mitochondrial proteins [1–4]. Conversely, *O*-GlcNAcase (OGA) reverses this abundant post-translational modification by removing the GlcNAc residue [5, 6]. *O*-GlcNAcylation regulates various cellular processes including transcription, translation, chromatin remodelling and cell cycle progression by modulating protein activity, stability, subcellular localization or protein–protein interaction [4, 7–10]. Moreover, a crosstalk can occur between *O*-GlcNAcylation and phosphorylation, either on adjacent sites or at the same sites of the target proteins [7, 9, 11–13]. Recently, the motif (pSp/T)P(V/A/T)(gS/gT) has been defined as a very specific and stringent phospho/*O*-GlcNAc crosstalk motif [14].

In mammalian cells, *O*-GlcNAcylation levels and the expression of OGT and OGA are highly regulated during the cell cycle, and disruption of *O*-GlcNAc cycling induces defects in cell cycle progression and mitosis [15–24]. The loss in *O*-GlcNAc homeostasis alters the expression of the early-induced transcription factors c-Fos, c-Jun, c-Myc and Sp1 [15] and the expression of cyclin D and p27^KIP1^ cell-cycle inhibitor [21, 25, 26]. It can also induce abnormal oscillations in the levels of cyclins E, A and B, resulting in aberrant mitotic-specific phosphorylation and defects in cytokinesis [12, 16–18, 23, 24]. To identify new targets of OGT during G1/S transition, we previously performed a differential analysis of the *O*-GlcNAcome of G1- and S phase synchronized human cells and identified the minichromosome maintenance (MCM) proteins MCM2/3/6/7 [22].

MCM2–7 complex is composed of six distinct MCM subunits assembled in a highly defined order: MCM5–MCM3–MCM7–MCM4–MCM6–MCM2. MCM proteins display a well-conserved organization of their functional domains, and possess an AAA+ ATPase activity in their C-terminal domain [27–29]. The heterohexameric MCM2–7 complex has a ring-shaped structure to encircle DNA, the MCM2–MCM5 interaction being identified as the gate that promotes the opening of the MCM2–7 complex [29, 30]. Sub-complexes containing MCM2/4/6/7, MCM4/6/7 or MCM3/5 have also been isolated from yeast and mammalian cells [31–36]. In vitro, the sub-assembly MCM4/6/7 exhibits the DNA helicase activity [37–40]. MCM2 which is weakly associated with this sub-complex negatively regulates its helicase activity [36, 37]. The strong interaction between MCM3 and MCM5 serves also negative regulatory function on MCM2–7 ATPase activity [36, 39, 41].

The MCM2–7 complex plays an essential role in the initiation of DNA replication which is a two-step process: licensing and firing. In G1 phase, MCM2–7 takes part in the formation of the pre-replicative complex (pre-RC) which is composed of ORC (origin replication complex, Orc1-6) and the licensing factors Cdc6 and Cdt1. During this licensing step, inactive head-to-head double MCM2–7 hexamers encircle DNA origins. As cells enter the S phase, the firing step requires the recruitment of Cdc45 and GINS to the MCM ring. Within the Cdc45/MCM2–7/GINS (CMG) complex, MCM2–7 exhibits the core replicative helicase activity necessary for parental DNA strands to unwind [29, 42, 43]. Finally, as cells progress in late S phase, MCM2–7 complexes progressively dissociate from chromatin to prevent DNA re-replication [44–47]. Importantly, most of the origins on which MCM2–7 complexes have been loaded constitute dormant origins that are not used normally in S phase. However, under conditions of replicative stress that induces replication fork stalling, excess MCM2–7 complexes license latent origins as a backup mechanism to prevent under-replication and maintain genome integrity [42, 48–51].

Phosphorylation of MCM subunits dramatically changes during cell cycle progression to finely regulate the functionality of MCM2–7 complex, from the licensing step to the dissociation of the complex from chromatin in late S phase [44, 52–62]. In this work, we investigate the role of *O*-GlcNAc post-translational modification on MCM2–7 complex. We show that all the MCM subunits are modified by OGT, mostly in the chromatin-bound fraction. We identify stable interaction between OGT and the MCM3, MCM6 and MCM7 proteins. Moreover, dysregulation of *O*-GlcNAc cycling by OGT silencing decreases the amount of chromatin-bound MCM2, MCM6 and MCM7 proteins and destabilizes MCM–MCM interactions.

## Materials and methods

### Antibodies, siRNA and chemicals

Thymidine (T1895), propidium iodide solution (P4864), RNAse A (R4875), complete protease inhibitor cocktail tablets and OGA inhibitor Thiamet G (ThG) were from Sigma-Aldrich (St Quentin Fallavier, France). ThG was used at 1 µM (prepared at 100 mM in DMSO) [63]. The OGT inhibitor acetyl-5S-GlcNAc (5S-G) was kindly provided by Pr. G. W. Hart and used at 50 µM (prepared at 50 mM in DMSO) [64]. GlcNAc was from TCI Chemicals (TCI Europe N.V., Belgium).

The following primary antibodies were used for Western blot: RL2 *O*-GlcNAc antibody (1:3000, ThermoScientific, Fisher Scientific, France), OGT (DM17 or Ti-14; 1:2000, Sigma-Aldrich), OGA (1:10,000, anti-MGEA5, ab124807, Abcam, Cambridge, UK), GST, MCM2 (PLA0060) and MCM5 (PLA0064) (1:3000, Sigma-Aldrich); MCM3 (N-19), MCM4 (H-300), MCM6 (H-8), MCM7 (141.2), GAPDH (0411) (1:3000, Santa Cruz, Heidelberg, Germany), Cyclin D1 (A-12), Cdt1 (H-300) (1:1000, Santa Cruz), Cdc6 (DCS-180) (1:1000, Merck, Darmstadt, Germany). The same antibodies were used for immunoprecipitation and indirect immunofluorescence or PLA experiments except for immunoprecipitation of MCM7 (D10A11, Cell Signaling Technology, Ozyme, Montigny-le-Bretonneux, France) and detection of MCM3 by PLA (3E1, Abgent, Euromedex, Souffelweyersheim, France). Normal control IgG polyclonal antibodies were used as negative controls for IP experiments (rabbit, mouse, or goat, Santa Cruz). The following secondary antibodies were also used: anti-goat IgG–HRP linked (1:30,000, Santa Cruz), anti-mouse IgG–HRP-linked and anti-rabbit IgG HRP-linked antibodies (1:10,000, GE Healthcare, V.W.R. Fontenay-sous-Bois, France), anti-rabbit IgG Alexa Fluor 488 and anti-mouse IgG Alexa Fluor 568 (1:600, ThermoScientific, Fisher Scientific, France). Control siRNA (siRNA univ. negative control) and siRNA against OGT (GGAGGCUAUUCGAAUCAGU[dT][dT] forward, ACUGAUUCGAAUAGCCUCC[dT][dT] reverse) were purchased from Sigma-Aldrich. siRNA against OGA (siGENOME Human MGEA5 (10724) siRNA SMARTpool) was from Dharmacon (GE Healthcare Europe GmbH, Velizy-Villacoublay, France). The p3XFLAG-OGT-siRNA-resistant vector was generated by directed mutagenesis using Phusion^®^ Hot start (NEB), p3XFLAG-OGT as temple and, 5′-agcagggaaaactgcaggaagctctgatgcattataaagaagcgatcaggatttcccctacctttgctgatgcctactc-3′ and 5′-gagtaggcatcagcaaaggtaggggaaatcctgatcgcttctttataatgcatcagagcttcctgcagttttccctgct-3′ as primers. Prior to transformation in DH5α, template was digested for 2 h at 37 °C by 1 U of DpnI (NEB). Positive clones were screened by sequencing.

### Cell culture, transfection and cell cycle synchronization

MCF7 and MDA-MB-231 breast cancer cell lines, and HEK 293T cells were routinely grown at 37 °C in a humidified atmosphere enriched with 5% CO_2_ in Dulbecco’s modified Eagle’s medium (DMEM) (Lonza, Basel, Switzerland) containing high glucose (4.5 g/L) and glutamine, and supplemented with 10% foetal calf serum (FCS) (Lonza) (complete medium).

For HA-OGT transfection, HEK293T cells were cultured in complete medium (8 × 10^5^ cells/100-mm dish) and when they reached around 60% of confluence, they were transiently transfected with HA-tagged OGT (1.25 µg/100-mm dish) using the Lipofectamine^®^ 2000 (Thermofisher, Fisher Scientific, France), according to the manufacturer’s instructions. HEK293T were harvested 48 h after transfection. For small interfering RNA (siRNA) transfection, MCF7 (1.5 × 10^6^ cells/100-mm dish) and MDA-MB-231 (10^6^ cells/100-mm dish) were reverse-transfected with Lipofectamine^®^ RNAiMAX (Thermofisher, Fisher Scientific, France) according to the manufacturer’s recommendations, using 60 pmol of siControl, siOGA or siOGT. Cells were harvested, respectively, after 60 or 72 h of transfection. To rescue OGT silencing, 1 day later siRNA transfection, MCF7 cells were transfected with the 3XFLAG-OGT-siRNA-resistant plasmid or the 3X-pCMV plasmid as the negative control (250 ng/w), using 2.5 µL Lipofectamine^®^ 2000. For both types of experiments (siRNA ± 3XFLAG-OGT-siRNA), when cell cycle synchronization was needed, cells were serum-starved 24 h after transfection and the synchronization protocol was followed, as mentioned below.

Cell cycle synchronization was performed using starvation followed by serum stimulation [22]. After 24 h in complete medium (DMEM-10% FCS), cell monolayer was rinsed with PBS (Lonza) and placed either 24 h in DMEM-0.5% FCS for MCF7 cells, or 48 h in serum-free medium for MDA-MB-231 cells. Cells were either harvested (time 0 h) or grown in complete medium to release cells in cell cycle. Cells were harvested at different time points, according to cell cycle progression that was systematically monitored by propidium iodide (PI) DNA staining and flow cytometry analysis, as previously described [22]. When mentioned, vehicle (DMSO, 1:1000), acetyl-5S-GlcNAc (5S-G, 50 µM) and ThG (1 µM) were added simultaneously with serum.

### Cell lysis and subcellular fractionation

After two washes of cellular layers with ice-cold PBS, whole cellular lysates (WCL) were obtained using RIPA buffer (150 mM NaCl, 50 mM Tris–HCl, pH 7.5, 1% (v/v) Triton X-100, 0.2% (w/v) NaDoc, 0.1% (w/v) SDS, containing 1 mM orthovanadate, 10 mM sodium fluoride and protease inhibitors) and placed on ice for 10 min. The lysate was then clarified by centrifugation at 18,800×*g* for 15 min and the supernatant was stored at − 20 or − 80 °C before use.

Subcellular fractionation was performed as previously described [55]. Cells were lysed in cytoskeleton extraction buffer (CSK) (100 mM NaCl, 10 mM PIPES, pH 7, 300 mM sucrose, 3 mM MgCl_2_, 0.1% (v/v) NP-40 with protease inhibitor cocktail and phosphatase inhibitors (10 mM NaF, 1 mM orthovanadate) at 4 °C for 20 min. Lysates were then centrifuged at 300×*g* for 5 min at 4 °C and supernatants were retrieved. A pellet wash was performed in CSK buffer, and after centrifugation at 300x*g* for 5 min at 4 °C, supernatants were retrieved. Combined supernatants were then clarified by centrifugation at 18,800×*g* for 15 min at 4 °C to constitute the soluble fraction (Sol.) containing the nucleocytoplasmic proteins. Finally, pellets were incubated in modified RIPA buffer (350 mM NaCl, 20 mM Tris, pH 7.5, 2.5 mM sodium pyrophosphate, 1% (v/v) Triton X-100) for 20 min on ice, sonicated for 3 min to break DNA, and then centrifuged at 18,800×*g* for 15 min at 4 °C. The clear supernatant was used as the chromatin-bound protein fraction (Chrom.).

### Immunoprecipitation and Western blotting

For co-immunoprecipitation experiments, non-SDS containing lysis and washing buffers were used, and chromatin-bound protein fraction (500 µg) was diluted ½ in NaCl-free modified RIPA-buffer to reduce NaCl concentration to 175 mM. Total, soluble and chromatin-bound protein extracts were precleared with a mix of protein A- and protein G-Sepharose beads (50:50) (GE Healthcare, V.W.R.) in lysis buffer for 2 h at 4 °C (20 µL/mg). After centrifugation (5 min, 5000×*g*), the supernatant was incubated with primary antibodies for 2 h or overnight at 4 °C (5 µg/mg). Then, protein A-Sepharose (for rabbit IgG) or protein G-Sepharose (for mouse and goat IgG) was added and incubated for an additional 1 h (30 µL/mg). Beads were washed successively three times with RIPA buffer (5 min), once in high-salt containing RIPA buffer (300 mM NaCl), and finally boiled in Laemmli buffer before separation by SDS-PAGE and transfer onto nitrocellulose membranes (Protran supported 0.45 µm NC, GE Healthcare).

For Western blot, membranes were blocked in 5% (w/v) nonfat dry milk in Tris-buffered saline (TBS 10×, Euromedex) with 0.05% (v/v) Tween 20 (TBS-T) and probed with primary antibodies overnight at 4 °C. The membranes were washed three times with TBS-T and incubated with the corresponding HRP-conjugated secondary antibody for 1 h at RT. Membranes were washed three times in TBS-T and immunoblots were developed with enhanced chemiluminescence (ECL prime Reagent, GE Healthcare, Supersignal West Pico Plus or Supersignal West Femto, ThermoScientific, Fisher Scientific, France). Image acquisition was done on a CCD camera (Fusion Solo, Vilber Lourmat, Marne-la-Vallée, France). The membranes were stripped in the antibody stripping buffer (Gene Bio-Application LTD, Euromedex, France) for 15 min at RT, extensively washed in water and TBS-T before reprobing with another antibody.

### sWGA lectin chromatography

Soluble and chromatin-bound proteins were enriched for GlcNAc-modified proteins using the GlcNAc-specific lectin succinylated wheat germ agglutinin (sWGA) immobilized on agarose (Vector Laboratories, Clinisciences, Nanterre, France) [65]. sWGA beads were first equilibrated in the twofold diluted RIPA-modified buffer used for chromatin-bound protein extraction (175 mM NaCl, 10 mM Tris, pH 7.5, 1.25 mM sodium pyrophosphate, 0.5% (v/v) Triton X-100). For each fraction (Sol. and Chrom.), the volume corresponding to 1 mg of proteins was adjusted to 500 µL in the initial lysis buffer, and then twofold diluted in PBS to get a final concentration of 1 mg/mL. Each fraction (1 mg) was incubated for 2 h at 4 °C with 50 µL of sWGA-beads. Beads were centrifuged at 1000×*g* for 3 min, and then washed under vigorous stirring successively, twice with 1 mL ½-diluted RIPA-modified buffer and twice with the same buffer containing 300 mM NaCl. Finally, beads were re-suspended in Laemmli buffer before heating at 95 °C for 7 min and SDS-PAGE. A negative control was performed for each fraction by adding 0.5 M free GlcNAc in the lysate before incubation with sWGA-beads.

### GST pull-down assay

Bacterial expression plasmids pGEX-2T for GST and GST–OGT fusion proteins were kindly provided by Drs. D. Leprince and X. Yang, respectively. For GST recombinant protein expression, BL21 DE-3 *Escherichia coli* were transformed with plasmids and cultured in LB medium containing 50 µg/mL ampicillin. When bacteria reached the exponential growth phase, induction was performed at room temperature with 0.1 mM IPTG for 4 h. Bacteria were centrifuged and pellets were resuspended in PBS containing a cocktail of protease inhibitors (Sigma-Aldrich). Crude lysates were obtained using the high-pressure homogenizer Emulsiflex-C3 (Avestin, Mannheim, Germany) and centrifuged at 10,400×*g* for 45 min. GST fusion proteins were immobilized on Glutathione Sepharose 4B beads (GE Healthcare) for 2 h at 4 °C under gentle agitation. Beads were successively washed for 5 min by gentle vortex in 20 mM Tris, pH 7.4, with 0.1% (v/v) Triton X-100 (twice) and in the same buffer containing 100 mM NaCl (twice), followed by centrifugation at 500×*g* for 5 min. For direct elution, beads were equilibrated twice in the elution buffer (50 mM Tris, pH 8, with 0.1% (v/v) Triton X-100) before adding 50 mM reduced glutathione (Sigma-Aldrich) in elution buffer. For GST pull down experiments using human cell lysates, 700 µg of proteins (soluble nucleocytoplasmic and chromatin-bound subcellular fractions) were added in each tube with the beads and incubated overnight at 4 °C with gentle agitation. Beads were successively washed three times in PBS with 0.1% Triton X-100, once in PBS with 0.1% Triton X-100 and 150 mM NaCl, and twice in 50 mM Tris, pH 8, with 0.1% Triton X-100 before elution as described before. Laemmli buffer was added in each eluted fraction, samples were boiled 5 min at 95 °C before SDS-PAGE.

### Click chemistry

We used the Click-It *O*-GlcNAc enzymatic labelling and the Click-It biotin glycoprotein systems (ThermoScientific, Fisher) to enrich *O*-GlcNAc proteins from cell lysates on streptavidin–agarose beads (Merck), as previously described [66]. Briefly, proteins from the chromatin-bound fraction were first precipitated using chloroform/methanol according to the manufacturer’s protocol, and then solubilized in the presence of 1% (w/v) SDS in 20 mM HEPES (pH 7.9). *O*-GlcNAcylated proteins were enzymatically labelled with a GalNAz residue using the Y289L galactosyltransferase (GalT) and UDP-GalNAz as the nucleotide sugar. A negative control (time point 18H) in which UDP-GalNAz was omitted, was carried out in parallel and treated exactly in the same conditions than samples. Labelled proteins were then subjected to a click chemistry reaction with a biotin–alkyne probe and enriched on streptavidin–agarose beads. After click-chemistry reaction, 10% of solubilized and Click-It-labelled proteins were removed to attest the presence of MCM proteins in labelled samples (Input Click-It).

We performed *O*-GlcNAc mass tag labelling to detect and quantify *O*-GlcNAcylated fraction of MCM proteins using a 4.4 kDa DBCO-PEG mass tag. After using the Click-It *O*-GlcNAc enzymatic labelling kit following the manufacturer’s instructions, 50 µg of GalNAz-labelled proteins were re-suspended in 1% SDS, 20 mM HEPES (pH 7.9) and incubated for 1 h at room temperature, under a gentle agitation, either with a 4.4 KDa DBCO-PEG at a final concentration of 10 mM (PEG+) or with DMSO (PEG−). Finally, labelled protein samples were precipitated using chloroform/methanol to remove excess of DBCO-PEG and boiled with Laemmli buffer before separation on polyacrylamide gel. For each MCM, electrophoretic Rf values were calculated to estimate the molecular weight of the non-PEGylated and the PEGylated shifted bands and establish how many *O*-GlcNAc sites are present on MCM proteins. Standard curve was established using the PageRuler Prestained Protein Ladder (10–170 kDa, ThermoScientific, Fisher). In vivo glycosylation stoichiometry was determined by quantifying the relative intensities of each band [67].

### Cell cycle and DNA synthesis analysis

Distribution of cells in G0/G1, S and G2/M was routinely determined by DNA staining with PI as previously described [22]. The rate of DNA synthesis was measured using the Click-iT^®^ EdU Flow cytometry assay kit (ThermoScientific, Fisher). After siRNA transfection or treatment with inhibitor and subsequent synchronization, cells were labelled with 10 µM EdU (5-ethynyl-2′-deoxyuridine) for 15 min before harvesting cells. Detection of EdU-positive cells was based on the click reaction (K+) with Alexa Fluor^®^ 647 (AF647) azide fluorescent dye according to the manufacturer’s recommendations. DNA content was further labelled with PI. Several controls were performed to set up the flow cytometry instrument parameters: no labelled cells, PI-labelled cells, Edu-K+-labelled cells and Edu-K−/PI-labelled cells. Cells were then analysed by flow cytometry on a CyAn ADP LX9 instrument using the Summit V4.3.04 software for data acquisition (Beckman Coulter, Life Sciences). AF647 and PI signals were read, respectively, in FL8 channel (laser 635 nm and *λ*_em_: 665/20 nm) and FL3 channel (laser 488 nm, *λ*_em_: 613/20 nm). Data were analysed using FlowJo software.

### Immunofluorescence and proximity ligation assay

MCF7 cells were grown on glass coverslips for 72 h and washed three times in cold PBS before fixation in 4% paraformaldehyde in PBS at room temperature for 20 min. After three washes in PBS (5 min per wash, at RT), permeabilization of cells was performed either with 0.5% (v/v) Triton X-100 in PBS for 2 min to detect OGT–MCM interaction [68], or 20 min with 0.5% (v/v) Triton X-100 in CSK buffer without NP-40 for the detection of MCM–MCM interactions [47]. This was followed by a quenching with 100 mM glycine (pH 7.4) in PBS for 20 min and three washes in PBS. Coverslips were incubated with blocking buffer (2% (v/v) FCS, 2% bovine serum albumin (w/v), 0.2% (w/v) gelatin in PBS) for 1 h at RT before incubation with the primary antibodies (1:100) diluted in the blocking buffer, overnight at 4 °C. For indirect immunofluorescence, coverslips were washed three times with 0.5% (v/v) Tween 20 in PBS and incubated with Alexa Fluor-conjugated secondary antibodies (1:600 in blocking buffer) for 1 h in the dark, at RT For the Proximity ligation assay (Duolink^®^ in situ kit, Sigma-Aldrich), after incubation with the primary antibodies and two washes in PBS, the coverslips were incubated with PLA PLUS and MINUS probes for mouse and rabbit, respectively, for 1 h, with the ligase for 30 min (ligation step), and with the polymerase for 2 h (amplification step, Duolink in situ detection reagents Green, *λ*_ex_/*λ*_em_: 495/527 nm) in a humidity chamber at 37 °C. Finally coverslips were washed three times in 0.5% (v/v) Tween 20 in PBS, once in PBS alone, and nuclei were stained with DAPI (50 µg/mL) for 2 min before mounting slides in Mowiol solution (Calbiochem, Merck chemicals, Nottingham, UK). Negative controls were done using only one of the primary antibodies. Immunofluorescence was detected through an inverted Zeiss LSM700 confocal microscope with a 40x oil immersion lens at room temperature and data were collected with the ZEN 2010 software (Zeiss, Oberkochen, Germany). Images from PLA were processed with ImageJ^®^ using a home-made plugin developed by TISBio to detect and quantify the nuclear fluorescent dots in labelled cells. Briefly, for each PLA file (channel 1, DAPI; channel 2, green PLA fluorescence), the nuclei were detected and labelled in channel 1 to define the r.o.i. (regions of interest) that were then applied to channel 2 to measure and quantify the fluorescence inside each labelled nucleus. The mean of fluorescence per cell is the ratio of the integrated density/area measured for each nucleus. Scatter dot plot (median with interquartile range) showing the mean of fluorescence per cell and statistical analysis (one-way ANOVA test, *p < *0.05*) were obtained using GraphPad Prism software.

## Results

### All the MCM subunits are *O*-GlcNAcylated when they are loaded onto chromatin

**Fig. 1:**
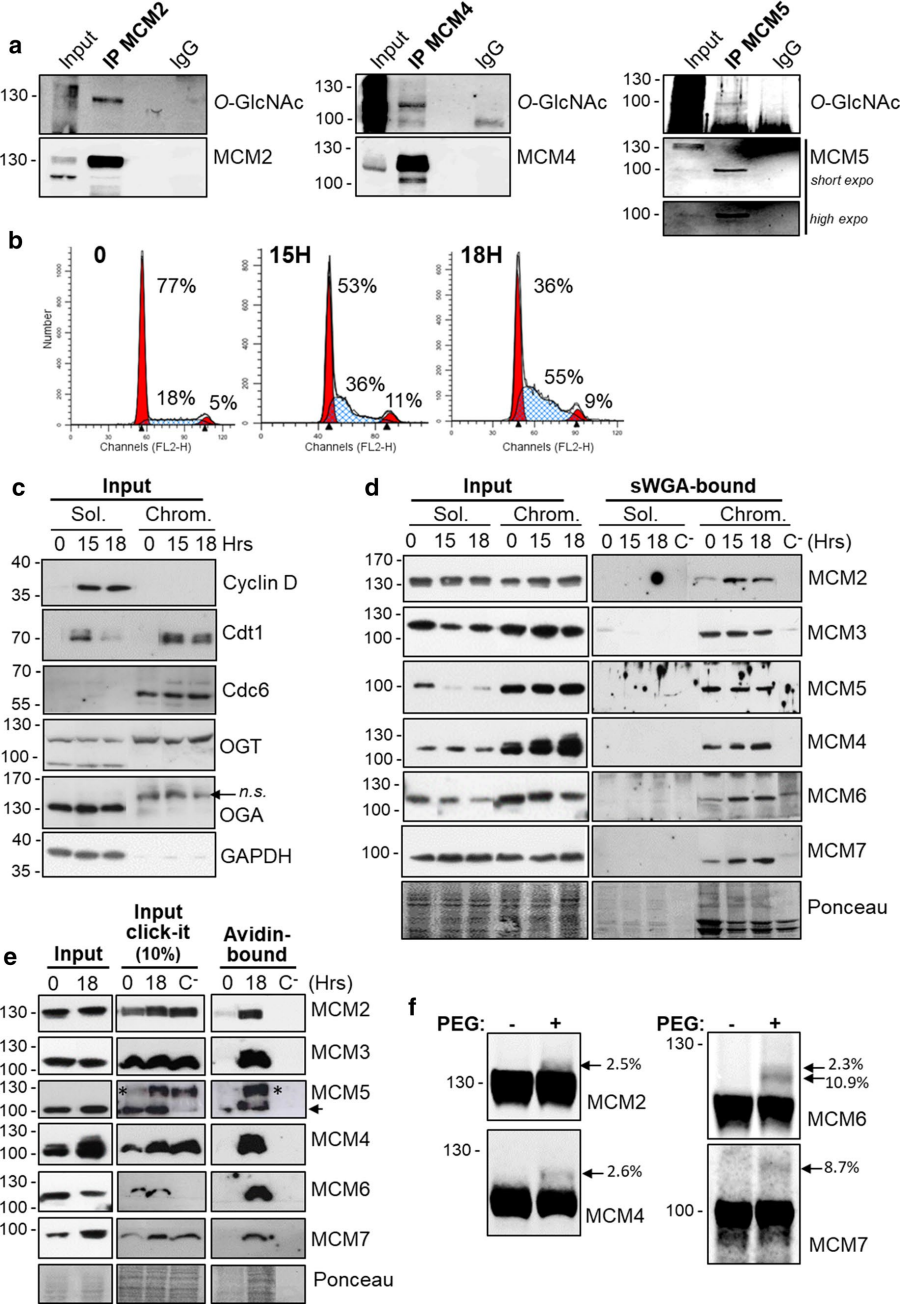
All MCM2–7 subunits are *O*-GlcNAcylated and mainly found in the chromatin-enriched fraction. **a** MCF7 cells were treated overnight with 1 µM ThG before lysis and immunoprecipitation of MCM2, MCM4 and MCM5 followed by Western blot analysis. Membranes were first incubated with anti-*O*-GlcNAc antibody (RL2), stripped and then reprobed with anti-MCM antibodies. **b** MCF7 cells were synchronized in cell cycle by starvation (time point 0) then released in S phase (15H, 18H). Cell cycle profiles were determined by FACS analysis after DNA staining with PI. Percentage of cells in G0/G1, S and G2/M phases are indicated. **c** The nucleocytoplasmic soluble fraction (Sol.) and chromatin-bound fraction (Chrom.) were obtained by subcellular fractionation of proteins from synchronized cells. Samples were analysed by Western blot for the indicated proteins (*n.s.* non-specific band). **d** *O*-GlcNAcylated proteins from soluble (Sol.) and chromatin-bound (Chrom.) fractions were enriched on sWGA-agarose beads. Incubation with excess of GlcNAc (0.5 M) was used as negative control (C^−^). MCM proteins were detected by Western blot before (Inp) and after enrichment on sWGA lectin (sWGA-bound). Equal loading was confirmed by Ponceau staining of the membranes. **e** *O*-GlcNAcylated proteins from chromatin-bound proteins of synchronized MCF7 cells were labelled with GalNAz and a biotin–alkyne probe (Input click-it) before enrichment on avidin–agarose beads (avidin bound). Negative controls were done by omitting UDP-GalNAz (C^−^). Samples were analysed by Western blot for MCM proteins before (Input) and after click-chemistry. (*) these bands correspond to the remnant signal for MCM2, despite membrane stripping. **f** *O*-GlcNAc-modified proteins from whole cell extract of MCF7 cells were enzymatically labelled with GalNAz and chemically modified with a 4.4 kDa DBCO-PEG mass tag (PEG+) or incubated with DMSO (PEG−) as negative control. MCM proteins were detected by Western blot and the number of *O*-GlcNAc sites and *O*-GlcNAcylation stoichiometry (indicated as the percentage of total MCM protein) were determined as reported in “Materials and methods”

We previously reported the *O*-GlcNAcylation of MCM3, MCM6 and MCM7 in MCF7 human cells after immunoprecipitation of endogenous MCM protein and detection by Western blot of the *O*-GlcNAc status using the anti-*O*-GlcNAc RL2 antibody [22]. Using the same approach, we show here that immunoprecipitated MCM2, MCM4 and MCM5 proteins are also *O*-GlcNAcylated in Thiamet G-treated MCF7 cells (Fig. 1a).

The phosphorylation status of MCM proteins regulates the chromatin loading of MCM2–7 complex [44, 53, 56, 57, 59, 61, 62, 69]. To investigate whether *O*-GlcNAcylated forms of MCM proteins are differentially distributed in the soluble and the chromatin-bound fractions during cell cycle progression, MCF7 cells were arrested in G0 by serum starvation and released in G1 phase by serum stimulation. Cells were harvested in early (15H) and late (18H) S phase, as measured by FACS (Fig. 1b). Subcellular fractionation from synchronized MCF7 cells was then performed to obtain a nucleocytoplasmic soluble fraction (Sol.) and a chromatin-bound fraction (Chrom.) [55]. Efficiency of fractionation was checked by Western blot using the G1-phase Cyclin D1 and GAPDH which are good markers for the soluble fraction (Fig. 1c). In contrast, as expected, the licensing factors Cdc6 and, to a lesser extent Cdt1, are exclusively detected in the chromatin-bound fraction [70, 71] (Fig. 1c). Interestingly, OGT is more abundant in the chromatin-bound fraction than in the soluble one, while OGA is only detected in the soluble one (Fig. 1c). Each MCM subunit was then detected by Western blot in the two subcellular fractions. Except for MCM2 and MCM7 which are equally detected in both fractions, the four other MCM subunits are preferentially located in the chromatin-bound fraction (Fig. 1d, Input). Then, to assess the *O*-GlcNAcylation of MCM proteins, we enriched *O*-GlcNAc proteins on succinylated WGA (sWGA)-agarose beads before Western blotting [65]. Negative controls in the presence of free GlcNAc were performed to confirm the specificity of the binding of GlcNAc-bearing proteins to the lectin (Fig. 1d, lanes C^−^). Although the six MCM subunits are present in both fractions, sWGA-bound MCM proteins were detected only in the chromatin-bound fraction (Fig. 1d). The same results were obtained with synchronized MDA-MB-231 cells (Suppl. Figure 1a–c). Our results demonstrate that *O*-GlcNAc-modified MCM proteins are stably loaded onto chromatin.

To ascertain that each chromatin-bound MCM subunit was individually and directly *O*-GlcNAcylated, we used a click chemistry approach to enrich and detect *O*-GlcNAcylated proteins from the chromatin fraction of quiescent and S phase-synchronized MCF7 cells. With this approach, SDS (1%) was used in the solubilization buffer, allowing dissociation and denaturation of protein complexes. *O*-GlcNAcylated proteins were enzymatically labelled with a GalNAz residue before the click chemistry reaction with the biotin–alkyne probe, allowing the enrichment of *O*-GlcNAcylated proteins on avidin–agarose beads [66]. The presence of MCM proteins in click-labelled samples was confirmed by the signal detected in the input (Input Click-It) (Fig. 1e). Western blotting of the avidin-bound proteins with anti-MCMs antibodies allowed us to confirm that all the six MCM2–7 subunits are strongly *O*-GlcNAcylated in the chromatin fraction of S phase-synchronized cells in comparison with quiescent cells (18H versus 0) (Fig. 1e).

Then to evaluate the number of *O*-GlcNAc site and the stoichiometry of *O*-GlcNAcylated isoforms of MCM proteins [67], we performed the chemoenzymatic labelling of *O*-GlcNAc proteins with UDP-GalNAz and GalT1 followed by the click reaction to conjugate a 4.4 kDa DBCO-PEG mass tag to the labelled glycoproteins. After SDS-PAGE separation, immunoblotting with MCM antibodies enabled the detection of both the non-glycosylated and glycosylated forms of MCM (indicated with arrows) from the chromatin fraction of asynchronous MCF7 cells (Fig. 1f). Using this approach, we were able to detect two sites on MCM2 and MCM4 (corresponding to a shift of an estimated MW of 10.1 ± 1.5 and 9.3 ± 1.1 kDa, respectively), the glycosylated forms representing less than 3% of both MCMs (Fig. 1f). For MCM6, we observed two distinct glycosylated species with two and three *O*-GlcNAc sites, representing 10.9 and 2.3% of MCM6 subunit, respectively. For MCM7, only one glycosylated form with three *O*-GlcNAc sites (ΔMW ≈ 12.3 kDa) was detected, representing nearly 9% of the protein. However, MCM proteins glycosylated at more than two or three sites may also exist in cells. They might be undetectable by immunoblotting due to steric hindrance of PEG molecules which could mask the epitopes and/or are stoichiometrically lower than the limit of detection.

### OGT stably interacts with several subunits of the MCM2–7 complex

**Fig. 2:**
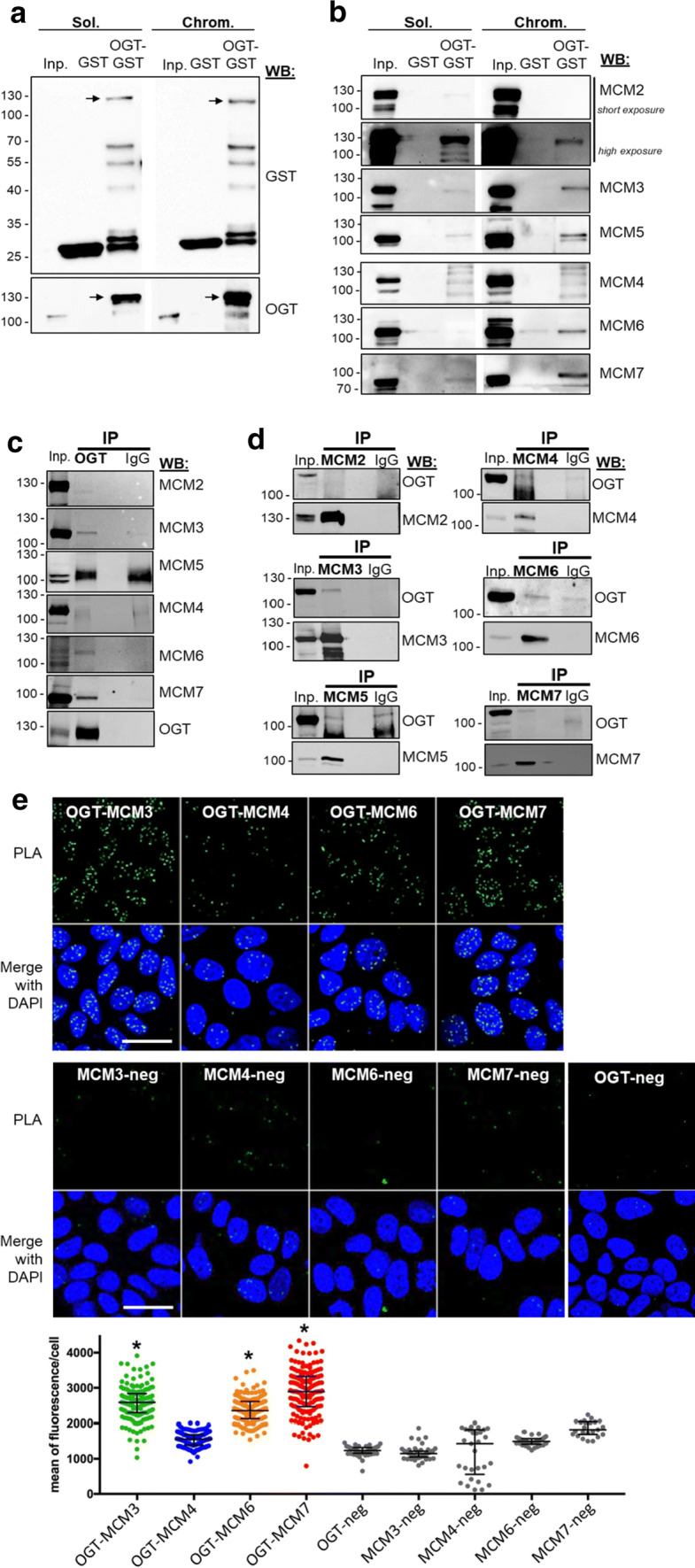
OGT stably interacts with several subunits of MCM2–7 complex. **a** GST pull-down assay using OGT–GST and GST (negative control) was performed using soluble (Sol.) and chromatin-bound (Chrom.) protein fractions from asynchronous MCF7 cells (Inp). Western blot analysis was performed using anti-GST and anti-OGT antibodies. **b** Eluted MCM proteins were detected by Western blot after GST pull-down assay. **c**, **d** OGT and subunits of MCM2–7 were immunoprecipitated from whole cell extracts of HEK293T cells transiently transfected with HA-OGT. Co-immunoprecipitated proteins were detected by Western blot using the indicated antibodies. **e** In situ proximity ligation assay was performed in fixed asynchronous MCF7 cells shortly permeabilized with 0.5% T-X100 in PBS to visualize interaction of endogenous OGT with MCM3, MCM4, MCM6 or MCM7. Nuclei were counterstained with DAPI and negative controls were performed by incubating fixed cells with only one of the primary antibodies (*MCM*-*neg* and *OGT*-*neg*). Quantification of PLA is presented as scatter dot plot; each dot represents the total signal of PLA in the nucleus of a single cell. Bars represents the median with interquartile range for each experience (one-way ANOVA test, *p < 0.05). Scale bar, 30 µM

The progressive and timely regulated assembly of MCM proteins with their partners is very important for the regulation of MCM2–7 stability and chromatin loading [29, 42]. To investigate whether OGT tightly interacts with the MCM2–7 subunits, we first performed a GST pull-down assay, using recombinant GST-tagged OGT immobilized onto Glutathione Sepharose beads as the bait [72]. Soluble and chromatin-bound extracts from asynchronous MCF7 cells were used as the source of prey proteins. The same experiment was conducted with GST as the negative control. After extensive washes and elution of proteins from the matrix with free glutathione, we first checked that GST and GST–OGT were efficiently eluted from Glutathione Sepharose beads using an anti-GST tag antibody, also confirmed by reprobing the membranes with an anti-OGT (Fig. 2a, lower panel). Then MCM proteins were detected by Western blot in the glutathione-eluted fractions; cellular extracts from both fractions were used as positive controls for the detection of endogenous MCM proteins (Fig. 2b, lanes Inp). Except for MCM4 that does not seem to bind to GST–OGT, both soluble and chromatin-bound MCM2/3/5/7 were able to interact with GST–OGT and not GST (Fig. 2b). For MCM6, we could observe a signal in the GST–OGT lane only in the chromatin-bound fraction. However, a faint band was also revealed in the negative control (GST alone), suggesting that MCM6 may also interact weakly with the GST tag in an unspecific manner (Fig. 2b). The same experiment was conducted with MDA-MB-231 cellular extracts in which we could observe the interaction between GST–OGT and MCM3, MCM5 and MCM7 in the two subcellular fractions (Suppl. Figure 1d). When we conducted the same experiment by adding 0.1% SDS in the MCF7 cellular extracts before incubation with beads, no signal was detected for any MCM in the GST–OGT lanes, indicating that SDS may denaturate the recombinant GST–OGT protein (data not shown). As we used here soft experimental conditions for the incubation, washing and elution buffers, we cannot exclude that eluted MCM proteins bind to OGT in an indirect manner due to the presence of MCM2–7 hexameric complexes and MCM proteins sub-complexes [31, 34, 37].

Then we conducted co-immunoprecipitation experiments to ascertain the interaction of OGT with MCM proteins in living cells. We first tried in MCF7 cells but we could not detect any co-immunoprecipitation of MCM proteins with endogenous OGT, corroborating the low OGT-bound/OGT-unbound MCM ratio that we observed using the GST pull-down approach (Fig. 2b). We used transitory OGT-transfected HEK293T cells and performed immunoprecipitation of either OGT or MCM from whole cell extracts. Western blot analysis showed that MCM3, MCM6 and MCM7 co-immunoprecipitate with OGT (Fig. 2c). Reverse IP allowed us to confirm that OGT interacts with MCM3 and MCM7 and, to a weaker extent, with MCM6 (Fig. 2d).

We next performed Proximity Ligation Assay (PLA) experiments in asynchronous cells, using antibodies against OGT, MCM3, MCM4, MCM6 and MCM7 for which we could validate their use in indirect immunofluorescence (data not shown). The specificity of PLA signal between OGT and MCM was confirmed by doing control experiments with only one of the primary antibodies followed by the incubation with both *minus* and *plus* PLA probes (Fig. 2e, lower panel). We observed strong PLA fluorescent signal in nuclei for OGT–MCM3, OGT–MCM6 and OGT–MCM7, in agreement with our GST pull-down and co-IP results (Fig. 2b–d). In contrast, the signal obtained for OGT–MCM4 was not significantly different from the MCM4-negative control (Fig. 2e), indicating that OGT does not stably interact with MCM4, as concluded by our co-IP results (Fig. 2c, d). It is important to note that we had to reduce the time of cell permeabilization to detect OGT–MCM interactions by PLA (2 min in 0.5% Triton X-100 instead of 20 min for the detection of MCM–MCM interactions by PLA, see Fig. 4b). This highlights that OGT is indirectly recruited to the chromatin via stable interaction with DNA-binding factors and chromatin effectors [4, 10, 72], while MCM proteins strongly associate with DNA [28, 29]. Altogether our results indicate that OGT is a new partner of MCM2–7 complex through its direct binding with MCM3, MCM6 and MCM7 subunits.

### *O*-GlcNAcylation does not affect MCM steady-state levels but impacts the loading of MCM2, MCM6 and MCM7 to the chromatin

**Fig. 3:**
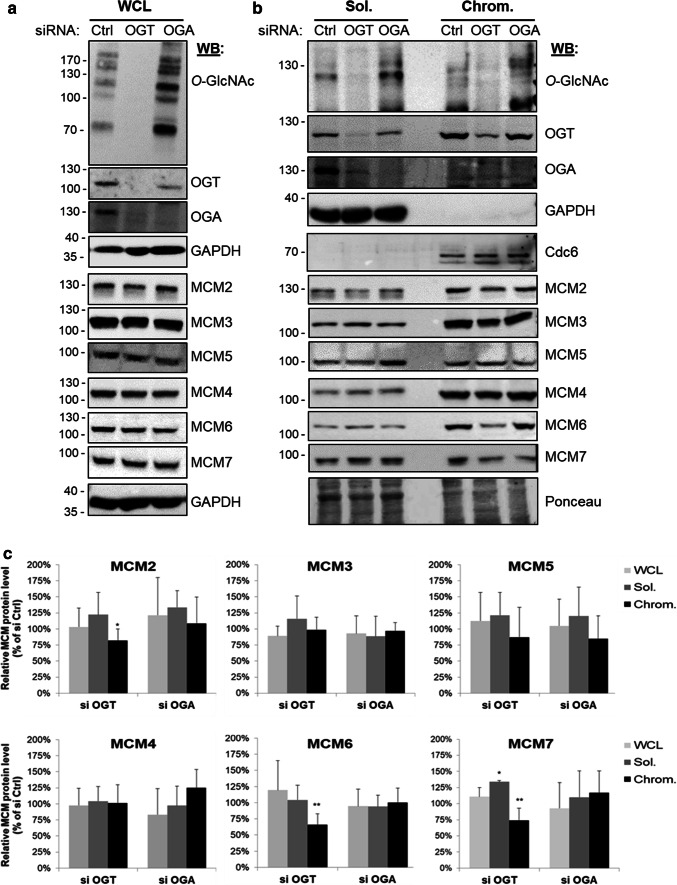
Down-regulation of OGT decreases the chromatin loading of MCM2, MCM6 and MCM7. MCF7 cells were treated with siRNA (Ctrl, OGT, OGA) for 60 h before harvesting. **a** The whole cellular extracts (WCL) and **b** the soluble (Sol.) and chromatin-bound (Chrom.) protein fractions were analysed by Western blot using anti-OGT, -OGA and *O*-GlcNAc proteins antibodies to confirm the efficiency of siRNA transfection. MCM proteins were also detected by Western blot using specific antibodies. Equal loading was confirmed using GAPDH antibodies (WCL) and Ponceau staining of the membranes (Sol./Chrom.). GAPDH and Cdc6 were used as markers for the soluble and chromatin fractions, respectively. **c** Band intensity was quantitated using Image J; the relative intensity of each MCM protein level in siOGT and siOGA conditions was normalized to that obtained for the siCtrl condition (100%) and depicted as a graph. Statistical analysis was performed by Student’s *t* test. Values are mean ± SEM of at least 4 independent experiments (**p < 0.05, *p < 0.1)

To test whether OGT and *O*-GlcNAc dynamics could regulate the binding of MCM proteins to chromatin, we induced silencing of either OGT or OGA by small interfering RNA (siRNA). Efficiency of OGT (siOGT) and OGA (siOGA) silencing was determined by Western blot against both enzymes and *O*-GlcNAcylated proteins (*O*-GlcNAc), and compared with random silencing (siCtrl) (Fig. 3a, b). As previously reported, OGT silencing induces a strong decrease in OGA protein level. Conversely, OGA silencing induces a moderate decrease in OGT protein level (Fig. 3a, b) [19, 73, 74]. Efficiency of the subcellular fractionation was attested by Western blot with anti-GAPDH antibody as a control for the soluble fraction, and anti-Cdc6 antibody for the chromatin-bound one (Fig. 3b). We did not observe any change in the expression of the six MCM2–7 subunits in the WCL when *O*-GlcNAcylation levels were disturbed (Fig. 3a–c), indicating that *O*-GlcNAc homeostasis may not regulate the steady-state level of MCM proteins.

Western blot analysis of soluble and chromatin-bound fractions showed that chromatin association of MCM3, MCM4, MCM5 was not affected by OGT or OGA silencing (Fig. 3b, c). In contrast, down-regulation of OGT slightly decreased the association of MCM6, MCM7, and to a lesser extent MCM2, with chromatin, while OGA silencing had no significant impact (Fig. 3b, c). For MCM7, this was accompanied by a moderate increase in the soluble fraction, suggesting that OGT may contribute to the loading or the stabilization of MCM7 onto chromatin. This hypothesis is reinforced by the strong interaction between OGT and MCM7 that we showed here for the first time (Fig. 2). We showed here a decrease in the chromatin loading of MCM2 when OGT was silenced, despite that no direct interaction between both proteins could be evidenced (Fig. 2c–e). MCM2 weakly interacts with the other MCM subunits and negatively regulates the helicase activity of the MCM4/6/7 sub-complex in vitro [31, 37, 40, 75]. Our results suggest that OGT might indirectly regulate the MCM2–7 complex via the regulation of MCM2–MCM interactions and the chromatin binding of MCM2.

### Perturbation of *O*-GlcNAc cycling destabilizes MCM2–7 complex

**Fig. 4:**
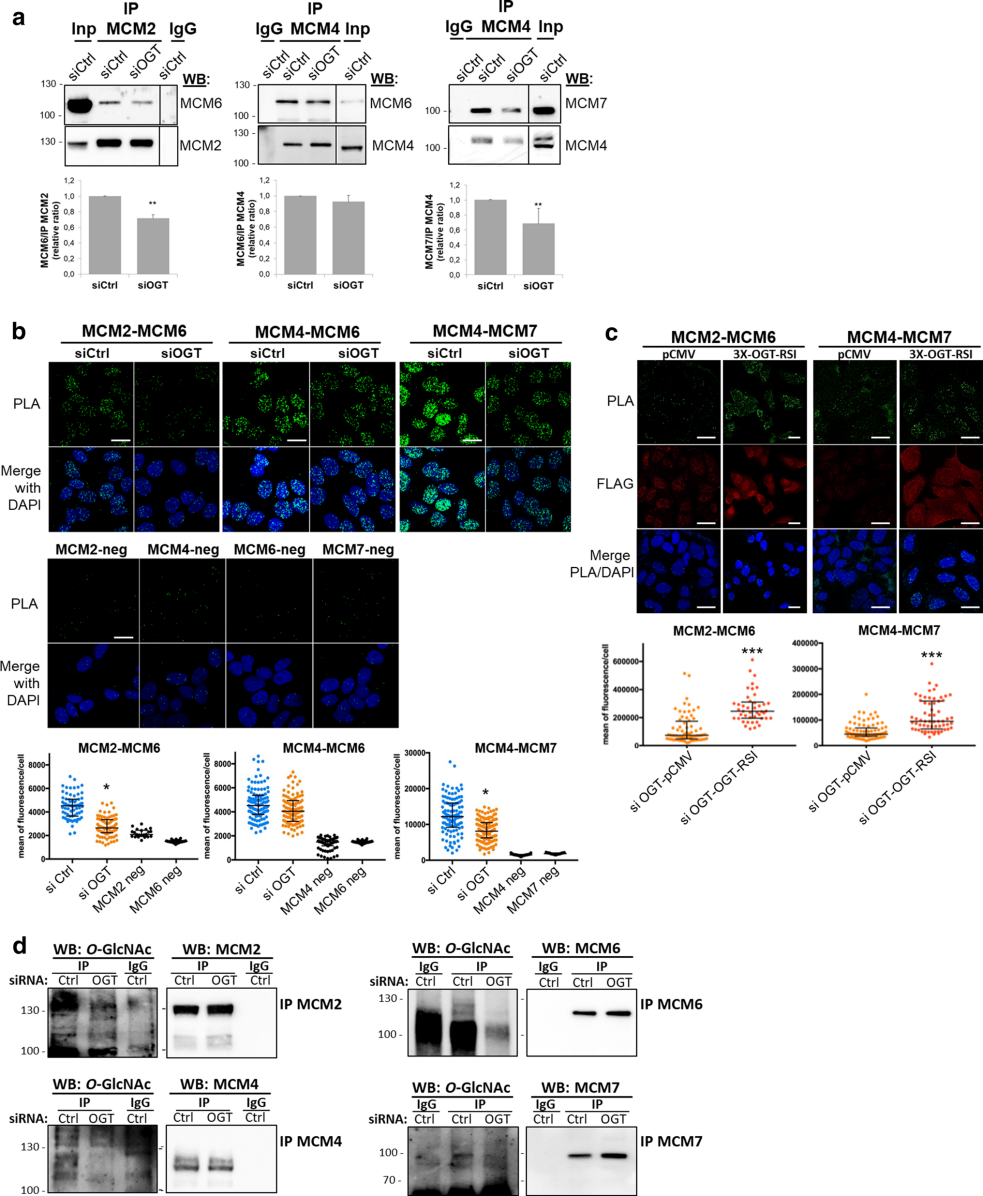
Silencing of OGT affects MCM2/MCM6 and MCM4/MCM7 interactions. MCF7 cells were transfected with siRNA (siCtrl, siOGT), then synchronized in S phase by stimulation with serum (18H) after serum starvation. **a** Chromatin-bound protein fraction was used for immunoprecipitation of MCM2 and MCM4 and Western blot analysis of co-immunoprecipitated MCM subunits. Band signal intensity was measured and statistical analysis was performed by Student’s *t* test. Values are mean ± SEM of three independent experiments (**p < 0.05). **b** MCM–MCM interactions were detected by in situ PLA and immunofluorescent confocal microscopy after nuclei counterstaining with DAPI. Quantification was performed as in Fig. 2e. Bars represents the median with interquartile range (one-way ANOVA test, *p < 0.05). Scale bar, 15 µM. **c** pCMV-3X or 3X-OGT-RSI plasmids were transfected in siOGT MCF7 cells for 24 h. Then cells were synchronized in S phase and MCM–MCM interactions were detected by in situ PLA, as described above (*t* test, ***p < 0.001). Scale bar, 20 µM. **d** MCM2, MCM4, MCM6 and MCM7 were immunoprecipitated from the chromatin-bound protein fractions and Western blots were revealed using the anti-*O*-GlcNAc antibody (RL2). Membranes were stripped and reincubated with the corresponding anti-MCM antibodies

The MCM2–7 helicase complex is a heterohexameric complex but sub-complexes containing MCM2/4/6/7, MCM4/6/7 or MCM3/5 have also been isolated in mammalian cells [31–33, 36]. Furthermore, the proper association of MCM subunits is essential for the establishment of active ATPase sites necessary for the helicase activity of the complex [27]. On the other hand, *O*-GlcNAcylation can alter protein–protein interactions [7]. Then we wondered whether MCM *O*-GlcNAcylation and/or OGT–MCM interaction could be involved in MCM–MCM interactions. To address this question, *O*-GlcNAc levels were reduced by OGT silencing, and co-IP and PLA experiments were performed to analyse MCM–MCM interactions in the chromatin-bound protein fraction (Fig. 4). OGT down-regulation induced a slight decrease of co-immunoprecipitation of MCM6 with MCM2, and MCM7 with MCM4 (Fig. 4a). In situ PLA confirmed these results: nuclear PLA signals between MCM2/MCM6 and MCM4/MCM7 were significantly lower in siOGT-transfected cells compared with those in siCtrl-transfected cells (Fig. 4b). In contrast, interaction between MCM4 and MCM6 tended towards a decrease in siOGT-cells, while differences in intensity of both co-IP and PLA fluorescence signals were not statistically significant between siCtrl and siOGT conditions (Fig. 4a, b, middle panels). To confirm that the decrease in MCM/MCM interactions that we observed in siOGT cells was due to the downregulation of OGT, we performed a rescue experiment by transfecting siOGT cells with a plasmid coding for a FLAG-tagged and siRNA-resistant OGT (3X-OGT-RSI). As shown in Fig. 4c, PLA signals obtained for both MCM2/MCM6 and MCM4/MCM7 were significantly restored when OGT was overexpressed in synchronized MCF7 cells, indicating that the presence of OGT may be important to maintain such interactions. To determine whether this could be related to a defect in *O*-GlcNAcylation of MCM proteins, we next performed immunoprecipitation of these four subunits from the chromatin-bound fractions of synchronized MCF7 cells and revealed their *O*-GlcNAc status by Western blot. Interestingly, the disturbance in MCM/MCM interactions upon OGT silencing is concomitant with a decrease of the *O*-GlcNAcylation level of MCM2, MCM4, MCM6 and MCM7 in the chromatin-bound fraction (Fig. 4d).

**Fig. 5:**
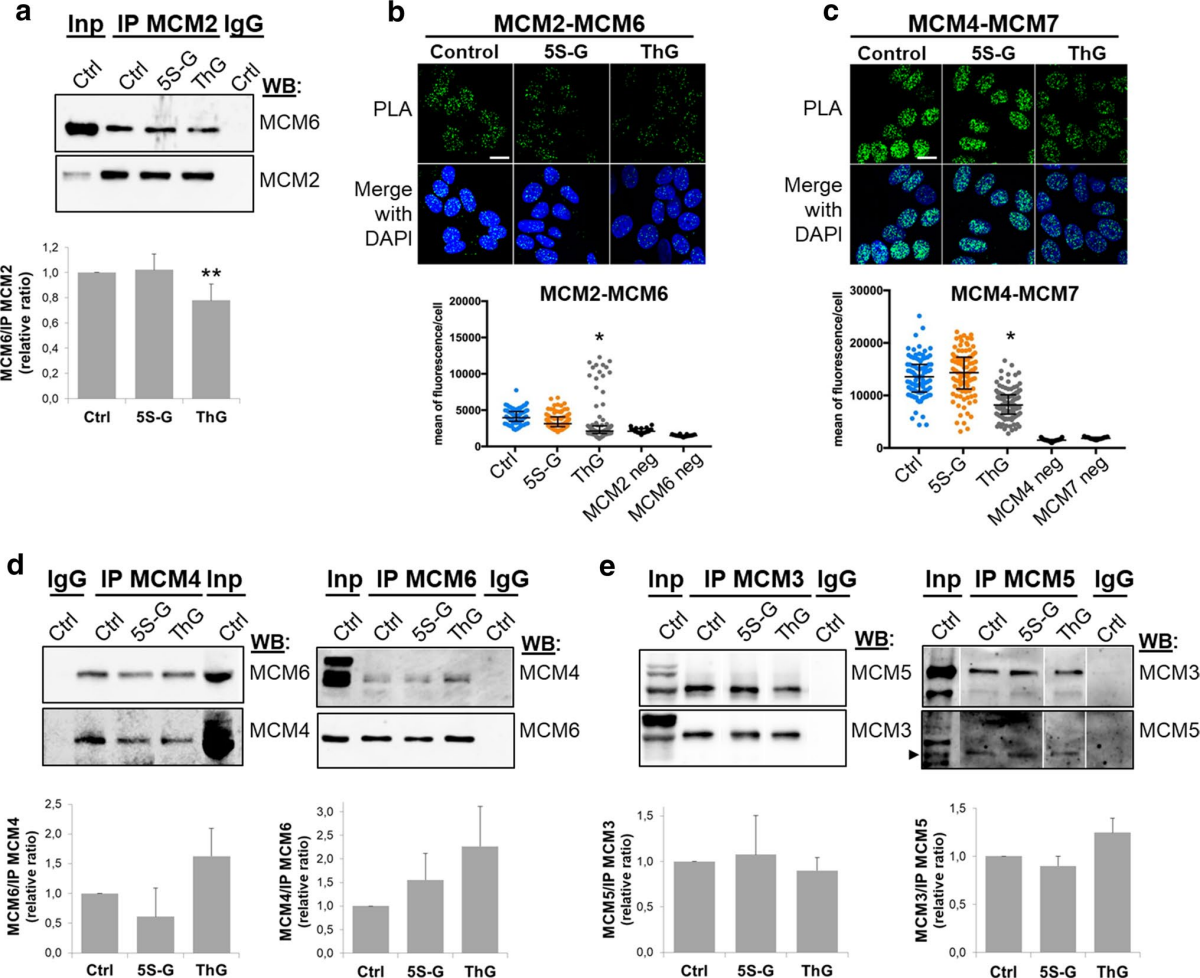
Hyper-*O*-GlcNAcylation induced by OGA inhibition alters MCM/MCM interactions. Serum-starved MCF7 cells were released in S phase by serum addition (18H), in presence of DMSO (Ctrl), 5S-G or ThG, before harvesting. **a**, **d** MCM proteins were immunoprecipitated from the chromatin-bound fraction and samples were analysed by Western blot using the indicated antibodies. Band signal intensity was measured and statistical analysis was performed by Student’s *t* test. Values are mean ± SEM of three independent experiments (**p < 0.05). **b**, **c** MCM–MCM interactions were detected by in situ PLA and immunofluorescent confocal microscopy. Nuclei were counterstained with DAPI. Quantification was performed as in Fig. 2e. Bars represent the median with interquartile range (one-way ANOVA test, *p < 0.05). Scale bar, 10 µM

Then we perturbed *O*-GlcNAc cycling in synchronized MCF7 cells using either 5S-G or ThG to inhibit the catalytic activity of OGT or OGA, respectively [63, 64] (Suppl. Figure 2). Surprisingly, we were not able to detect any significant and reproducible changes in MCM2/MCM6 and MCM4/MCM7 interactions in 5S-G-treated cells, as shown by co-IP and PLA experiments (Fig. 5a, b). However, both approaches showed a moderate decrease in MCM2/MCM6 interaction in the chromatin-bound fraction when *O*-GlcNAc levels were increased through OGA inhibition by ThG treatment (Fig. 5a, b). It is important to note that we could not detect MCM2 when MCM6 was immunoprecipitated (data not shown), probably due to the weak association of MCM2 with MCM6 [40]. Moreover, MCM4/MCM6, MCM4/MCM7, and MCM3/MCM5 interactions did not seem to be sensitive to OGT inhibition (Fig. 5c–e). Co-immunoprecipitation of MCM3 and MCM5 was also not significantly perturbed by ThG (Fig. 5e). However, PLA approach shows that inhibition of OGA induced a moderate decrease in MCM4/MCM7 interactions (Fig. 5c), whereas it tended to increase MCM4/MCM6 co-immunoprecipitation albeit in a non-significant manner (Fig. 5d), that we could confirm by in situ PLA (data not shown).

### Effect of perturbation of *O*-GlcNAc cycling on S phase progression and DNA replication

We next determined whether dysregulation in *O*-GlcNAc homeostasis would affect DNA replication and S phase progression. We used synchronized MCF7 cells that were either transfected by siRNA or treated with potent inhibitors of OGT and OGA. After serum stimulation, cells were harvested at different time points and cell cycle progression was analysed by flow cytometry after staining with PI. DNA replication rate was also evaluated by labelling nascent DNA within the last 15 min of serum stimulation with EdU. After click chemistry reaction and PI staining, the percentage of EdU-positive cells was determined by flow cytometry. In parallel, cells were seeded and treated with either siRNA or inhibitors, and counted every day during 5 days.

For each experiment, the efficiency of treatments was systematically confirmed by Western blotting (Suppl. Figure 3). It is noteworthy that the decrease in *O*-GlcNAcylation levels induced by 5S-G was accompanied by a significant decrease in OGA expression, whereas elevation of *O*-GlcNAcylation levels induced by ThG treatment was accompanied by a decrease in OGT expression and an increase of OGA protein (Suppl. Figure 3a, see also Suppl. Figure 2). Similarly, as we observed above (Fig. 3), silencing of OGT strongly decreased OGA protein level. Conversely silencing of OGA decreased the levels of OGT, albeit to a lesser extent (Suppl. Figure 3b).

**Fig. 6:**
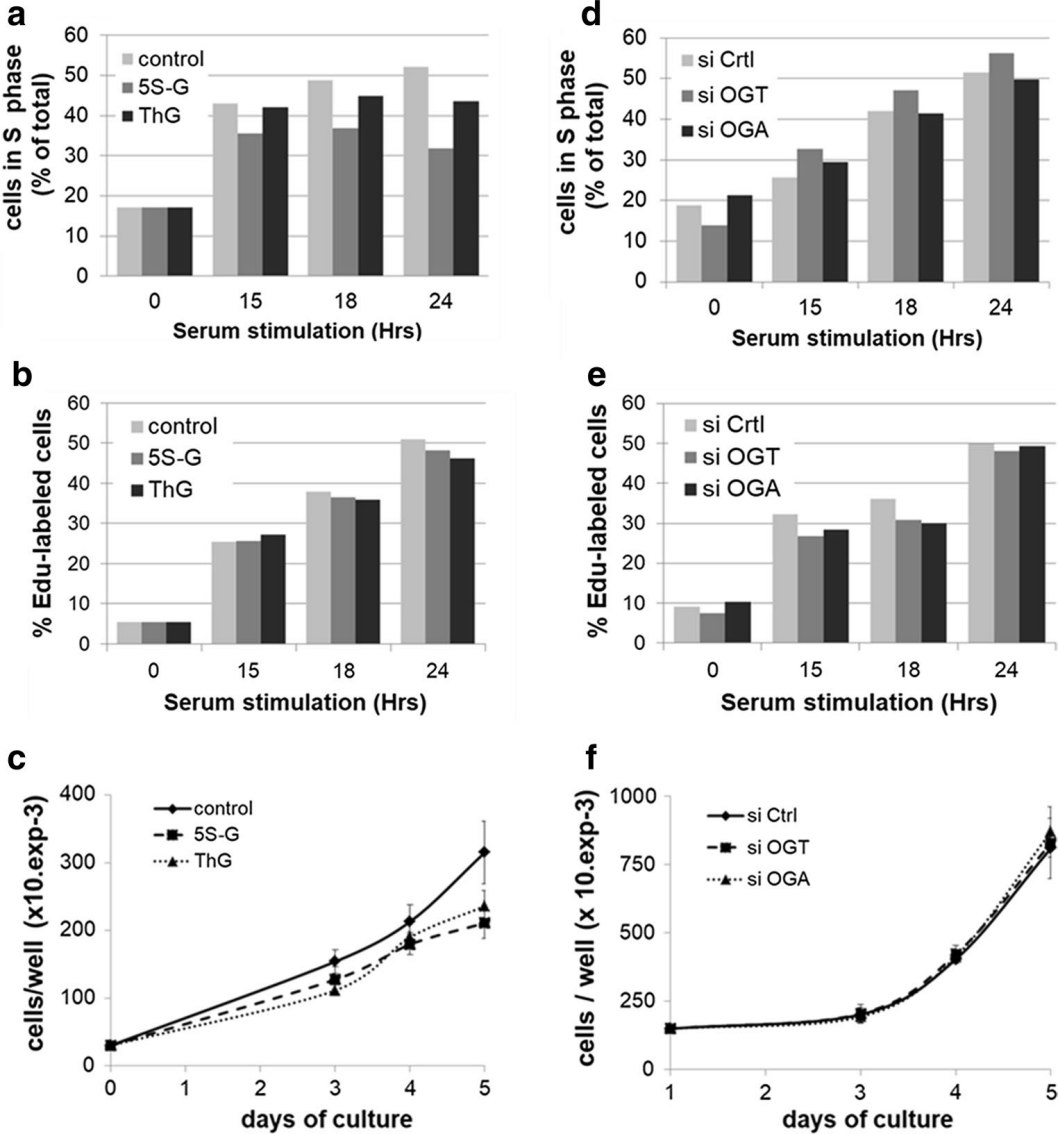
Perturbation of *O*-GlcNAc cycling does not affect DNA replication rate in MCF7 cells. Serum-starved MCF7 cells were released into the cell cycle by serum addition for the indicated times in the presence of OGT and OGA inhibitors (5S-G, ThG) or DMSO (Ctrl). **a** Cell cycle distribution was determined by flow cytometry after DNA staining with PI. Percentage of cells in S phase is indicated for each time point. Results are representative of three independent experiments. **b** EdU was added during the last 15 min of serum stimulation to label nascent DNA using the Click-It EdU flow cytometry assay, and DNA content was stained with PI before flow cytometry analysis. Percentage of EdU-positive cells is indicated for each time point. Results are representative of three independent experiments. **c** One day after seeding, MCF7 cells were treated with DMSO (Ctrl), 5S-G or ThG. Cells were counted every day for 5 days and medium was replaced every 2 days (with inhibitors). Values are mean ± SEM of three independent experiments. **d** MCF7 cells were transfected with siRNA (siCtrl, siOGT, siOGA) and then synchronized as in **a**. Results are representative of three independent experiments. **e** MCF7 cells were treated as above. Percentage of EdU-positive cells was obtained as in **b**. Results are representative of three independent experiments. **f** MCF7 cells were transfected with siRNA (siCtrl, siOGT, siOGA). Cells were counted every day for 5 days and medium was replaced every 2 days (with siRNA). Values are mean ± S.E.M. of three independent experiments

As shown in Fig. 6a, inhibition of OGT by 5S-G induced a moderate decrease in the percentage of MCF7 cells in S phase compared with control condition (35.6 versus 43% at 15H; 32 versus 52% at 24H). However, this was not correlated with changes on the rate of EdU incorporation (Fig. 6b), although the cellular growth of 5S-G-treated cells was significantly reduced by 30% after 5 days of treatment (Fig. 6c). Likewise, inhibition of OGA did not modify the DNA replication rate but tended towards a slowdown in S phase progression; this was accompanied with a decrease of 25% of proliferation after 5 days of treatment (Fig. 6a–c). Surprisingly, S phase progression of siOGT- and siOGA-transfected MCF7 cells was not significantly affected compared with that of control siRNA-transfected cells, nor was the rate of DNA replication (Fig. 6d, e). In agreement with these results, no difference in cellular growth was observed between siCtrl, siOGT and siOGA conditions in MCF7 cells (Fig. 6f).

## Discussion

The heterohexameric MCM2–7 helicase complex is crucial for the initiation of DNA replication and is finely regulated by post-translational modifications, including phosphorylation. We previously showed that MCM3, MCM6 and MCM7 were *O*-GlcNAcylated by OGT [22]. Here we demonstrate that MCM2, MCM4 and MCM5 are also *O*-GlcNAcylated in human cells (Fig. 1a, b). This result is strengthened by two recent works which have identified MCM2, MCM3, MCM4 and MCM5 as *O*-GlcNAc-modified proteins using metabolic incorporation of chemical GlcNAc analogue probes combined with click chemistry labelling [76, 77]. Using a mass-tagging strategy, we show that the *O*-GlcNAcylated MCM proteins are of low stoichiometry (from less than 3% for MCM2 and MCM4, to 13% for MCM6) (Fig. 1f), as it has been evidenced for many *O*-GlcNAc-modified proteins [78, 79]. We could detect 2 *O*-GlcNAc sites on MCM2 and MCM4, 2 and 3 *O*-GlcNAc sites on MCM6, and 3 *O*-GlcNAc sites on MCM7, although we cannot exclude that some of the *O*-GlcNAc sites may not have been detectable using this approach. Many phosphorylation sites have been characterized on MCM proteins, especially within the N-terminal extension of MCM2 and MCM4; some of them are known to play crucial roles in regulating MCM2–7 loading and helicase activity [53–58, 60–62]. It is tempting to speculate that *O*-GlcNAc modification may cooperate or compete with phosphorylation to regulate MCM2–7 complex. Therefore, we performed the in silico analysis of the potential *O*-GlcNAc/phosphorylation crosstalk according to the recently published stringent motif (S/T)-P-(V/A/T)-(gT/gS) [14]. We also used the YinOYang1.2 server to predict the *O*-GlcNAc sites on MCM proteins, by taking into account only the high-scoring potential *O*-GlcNAc sites (http://www.cbs.dtu.dk/services/YinOYang). Such prediction tool has to be taken with caution and experimental data are required to ascertain their localization. Using the human primary sequences of MCM2 to MCM7 proteins of the UniProtKB database, we found the specific crosstalk motif [^611^SPVT^614^] within the sensor 2 subdomain of MCM3, which is adjacent to the potential predicted *O*-GlcNAc site at Thr610. It is noteworthy that phosphorylation of Ser611 of MCM3 has been identified by mass spectrometry in human leukaemia cells, but to date, no functional role has been assigned to this residue [80]. For MCM4, we found the motif [^3^SPAS^6^] in the N-terminal extension of which is known to be phosphorylated at multiple sites (http://www.PhosphoSitePlus) [53, 56]. Interestingly, three highly potential *O*-GlcNAc sites are located within the same N-terminal region of MCM4, at position Ser2, Ser3 and Thr7, suggesting that the N-terminal tail of MCM4 may be targeted by both kinases and OGT to regulate MCM2–7 helicase complex. On the other hand, for MCM5 subunit, we found the degenerated crosstalk motif [^133^SPSS^136^], which is located at the hinge of two structural subdomains of the N-terminal domain (NTD) of MCM5, NTD-A (A subdomain of NTD, amino acids 32–129) and oligonucleotide/oligosaccharide-binding (OB)-fold (amino acids 136–172) (http://www.ebi.ac.uk/interpro/InterPro) [28]. It is of interest that the two predicted *O*-GlcNAc sites of MCM5, Ser135 and Ser136, are adjacent to Ser133 which is a phosphorylation site with unknown function [81]. Although no such *O*-GlcNAc-phospho crosstalk motif has been found for MCM2, the potential *O*-GlcNAc site Thr25 is close to Ser27 which is phosphorylated by Cdc7/Dbf4 and takes part in the initiation of DNA replication [54, 59]. This in silico analysis strongly suggests that a crosstalk may occur between *O*-GlcNAcylation and phosphorylation on MCM proteins, adding a layer of complexity to finely regulate MCM2–7 complex. Further mass spectrometry analysis is now required to unambiguously identify the glycosylation sites of human MCM proteins and experimentally test this hypothesis.

Our results show that OGT glycosylates the six subunits of MCM2–7 complex when quiescent cells progress normally in S phase, and that *O*-GlcNAcylated MCM proteins are present nearly exclusively in the chromatin fraction (Fig. 1d, e). This indicates that *O*-GlcNAcylation of MCM2–7 complex may occur mostly when MCM proteins are loaded onto chromatin. Another possible explanation to the lack of *O*-GlcNAc-enriched MCM proteins in the soluble fraction is that the turnover of MCM protein *O*-GlcNAcylation may be higher in the nucleoplasmic soluble fraction in which both OGT and OGA were present, than in the chromatin-bound fraction in which we could not detect OGA (Figs. 1c, 3b, Suppl. Figure 2). *O*-GlcNAcylation of MCM proteins might help in the recruitment of MCM2–7 complex onto the chromatin, as described for the phosphorylation of MCM2 and MCM3 [54, 60]. In contrast, for MCM4, *O*-GlcNAc modification could act in an opposite manner to phosphorylation, since highly phosphorylated MCM4 is less tightly bound to chromatin than underphosphorylated form of MCM4 [31].

On the other hand, we demonstrate that OGT strongly interacts with distinct MCM2–7 subunits in human cells. Indeed, biochemical and in situ approaches show that OGT is a new partner of MCM2–7 complex through its direct binding with MCM3, MCM6 and MCM7 subunits (Fig. 2). Our findings are in agreement with previous studies that demonstrate that the recruitment of MCM2–7 partners occurs via their specific interaction with one or two MCM subunits, as it has been reported for the interactions of the licensing factor Cdt1 with MCM6 [82] and Cdc45 with MCM2 and MCM5 [43]. It has also been shown for the DDK subunits: whereas Dbf4 strongly binds to MCM2, Cdc7 interacts with both MCM4 and MCM5 subunits [83]. Our data suggest that OGT might regulate the chromatin loading of MCM6 and MCM7 through this strong interaction. Indeed, down-regulation of OGT protein level by siRNA decreases the chromatin-bound level of MCM6 and MCM7 (Fig. 3b). This hypothesis is reinforced by the destabilization of MCM2/MCM6 and MCM4/MCM7 interactions when OGT is silenced in synchronized MCF7 cells (Fig. 4a–c). Given the fact that we did not observe significant changes in these interactions when OGT is inhibited by 5S-G (Fig. 5a–c) whereas OGT rescue experiment in siOGT-transfected cells could restore them (Fig. 4c), we hypothesize that in normal culture conditions, OGT protein might be more important than its catalytic activity and act as a scaffold protein to regulate such interactions and recruit MCM2–7 complexes to the chromatin through its direct binding to MCM6 and MCM7 subunits. Moreover, our data show that the destabilization of the interactions between MCM2/MCM6 and MCM4/MCM7 are concomitant with a decrease in the *O*-GlcNAcylation levels of these MCM subunits (Fig. 4d). This suggests that *O*-GlcNAc modification of MCM proteins may be also involved in the stabilization of MCM2–7 complex onto chromatin. Since this glycosylation is known to stabilize protein–protein interactions [4, 6], we would have expected an elevation of MCM/MCM interactions upon OGA inhibition. But it did not, since the increase of the overall *O*-GlcNAcylation levels in ThG-treated cells slightly downregulated the binding of MCM2/MCM6 and MCM4/MCM7 in S phase-synchronized MCF7 cells (Fig. 5b, c). Altogether, our data suggest that *O*-GlcNAc homeostasis might contribute to stabilize MCM–MCM interactions. Further investigations are needed to decipher the molecular mechanisms underlying the regulation of the chromatin loading and stabilization of MCM2–7 complex through the recruitment of OGT and *O*-GlcNAcylation of the MCM2–7 complex subunits.

Here we perturbed *O*-GlcNAc cycling using either siRNA or selective and potent inhibitors of the *O*-GlcNAc enzymes, OGT and OGA. As previously reported, in case of disruption to *O*-GlcNAc homeostasis, cells adjust OGT and OGA expression to compensate for the changes in *O*-GlcNAc levels, with OGA protein expression being more sensitive to *O*-GlcNAc homeostasis than OGT. Reduction in *O*-GlcNAc levels induces a decrease in OGA protein level and reciprocally, elevation of *O*-GlcNAcylation induces a decrease in the expression of OGT, (Fig. 3, Suppl. Figures 2, 3) [16, 19, 22, 64, 73, 74, 84–86]. Similar results were obtained not only in MDA-MB-231 breast cancer cells, but also in the colorectal cell lines HCT116, HT29 and CCD841CoN (unpublished data). This mutual regulation of OGT and OGA to compensate the loss of *O*-GlcNAc homeostasis is far to be fully understood. To date, no mechanism has been established that can explain how OGA protein is as much downregulated when OGT is inhibited or silenced. However, Zhang and collaborators have shown that Thiamet G increases the transcription of OGA mRNA [84]. Moreover, it has been recently demonstrated that OGT mRNA levels are controlled by an intron splicing silencer (ISS) that induces the nuclear degradation of the mRNA under high *O*-GlcNAcylation conditions, thus allowing a decrease in OGT protein levels [85]. This may explain the lower levels of OGT that we and others have observed upon inhibition or silencing of OGA. In contrast, an overall decrease of *O*-GlcNAcylation upon OGT inhibition induces an efficient splicing of OGT mRNA to produce a cytoplasmic mRNA that will be further translated [85].

Neither silencing nor inhibition of the *O*-GlcNAc-regulating enzymes perturbs significantly the rate of DNA synthesis in synchronized human MCF7 cells (Fig. 6b, e). Nevertheless, the S phase progression and growth rate slow down when OGT is inhibited and to a lesser extent when OGA is inhibited (Fig. 6a, c). Thus, the delay in S phase entry induced by OGT or OGA inhibition may not be related to a defect in DNA synthesis in MCF7 cells. It could be due to an abnormal activity or expression of cell cycle-related proteins that are known to be directly or indirectly regulated by OGT, like transcription factors, Cyclin/CDK, or cell cycle inhibitors [15, 21, 22, 87]. Collectively, our results indicate that the effects of OGT down-regulation on MCM proteins that we report here for the first time are not sufficient to disrupt S phase progression and DNA synthesis in human MCF7 cells. Although we assessed the DNA helicase activity in an indirect manner through the measurement of Edu incorporation, our results are consistent with previous work demonstrating that an acute down-regulation of each one of the MCM2–7 subunits by silencing approach does not slow down the replication fork speed during DNA elongation in human cells [50]. Therefore, we believe that when cells normally progress in S phase, perturbation of *O*-GlcNAc cycling may destabilize MCM/MCM interactions but without interfering with MCM2–7 helicase activity and DNA replication. The possible explanation is that a large excess of MCM2–7 complexes is loaded to chromatin during G1 phase to license dormant replication origins that are not used during normal DNA replication but are required under conditions of replicative stress to maintain genome integrity [48–50]. This hypothesis is reinforced by recent studies highlighting that OGT relocates to the sites of DNA damage and targets key signalling proteins and DNA polymerase η in response to DNA damage [13, 88, 89]. In conclusion, our work demonstrates that OGT is a new partner of MCM2–7 complex and regulates MCM–MCM interactions. Although further investigations are needed to investigate the molecular mechanisms in detail, it opens up new prospects for the role of OGT and *O*-GlcNAc post-translational modification in the regulation of DNA replication under conditions of unperturbed replication as well as replicative stress.

### Electronic supplementary material

Below is the link to the electronic supplementary material.

**Supplementary Figure Captions: Suppl. Figure** **1** *O*-GlcNAcylated MCM2-7 subunits are mainly found in the chromatin-enriched fraction in MDA-MB-231 cells. Serum-starved MDA-MB-231 cells were released into the cell cycle by serum addition for the indicated times. **a** Cell cycle distribution was determined by flow cytometry after DNA staining with PI. Percentage of cells in G0/G1, S and G2/M phases are indicated. **b** The nucleocytoplasmic soluble (Sol.) and chromatin-bound fractions (Chrom.) were analysed by Western blot for the indicated proteins. **c** *O*-GlcNAcylated proteins from both fractions were enriched on sWGA-agarose beads. Incubation with excess of GlcNAc (0.5 M) was used as negative control (C^−^). MCM proteins were detected by Western blot before (Input) and after enrichment on sWGA lectin (sWGA-bound). **d** Recombinant GST-tagged OGT (OGT–GST) and GST alone (GST) were immobilized onto Glutathione Sepharose beads. GST pull-down assay was performed using soluble (Sol.) and chromatin-bound (Chrom.) protein fractions from asynchronous MDA-MB-231 cells (Inp). Eluted MCM proteins were detected by Western blot and anti-GST antibodies were used to confirm the presence of the recombinant proteins after elution. **Suppl. Figure** **2** Efficiency of inhibition of OGT and OGA in MCF7 cells. Serum-starved cells were released in S phase by serum addition for 18 h in presence of DMSO (Ctrl), 5S-G (50 µM) or ThG (1 µM), before harvesting. Proteins from subcellular fractions (Sol. and Chrom.) were separated by SDS-PAGE and analysed by Western blot for the indicated proteins. Equal loading was confirmed by Ponceau staining of the nitrocellulose membrane. **Suppl. Figure** **3** Serum-starved MCF7 were released in S phase by serum addition for the indicated times. **a** DMSO (1/1000, Ctrl), 5S-G (50 µM) or ThG (1 µM) was added at the same time as serum. **b** cells were transfected with siRNA 24 h before serum starvation. Whole cell lysates were resolved by SDS-PAGE and analysed by Western blot for the indicated proteins. (PPTX 1094 kb)
