# Supplementary figures and images for: O-GlcNAc transferase associates with the MCM2–7 complex and its silencing destabilizes MCM–MCM interactions
Source: Cell Mol Life Sci. 2018 Aug 1;75(23):4321–39. doi: 10.1007/s00018-018-2874-0 (PMC6208770; doi:10.1007/s00018-018-2874-0)

## Slide 1
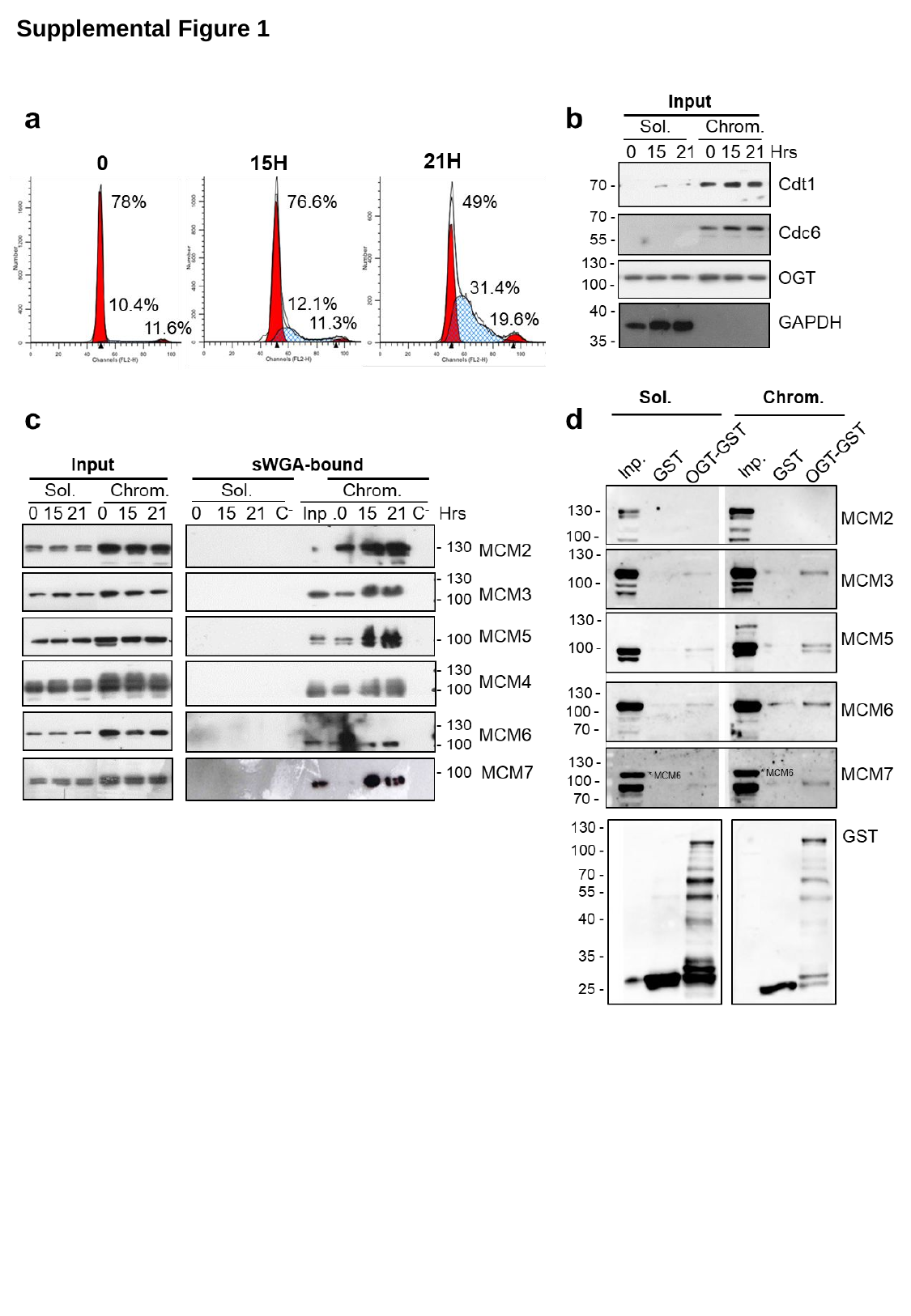

Supplemental Figure 1

## Slide 2
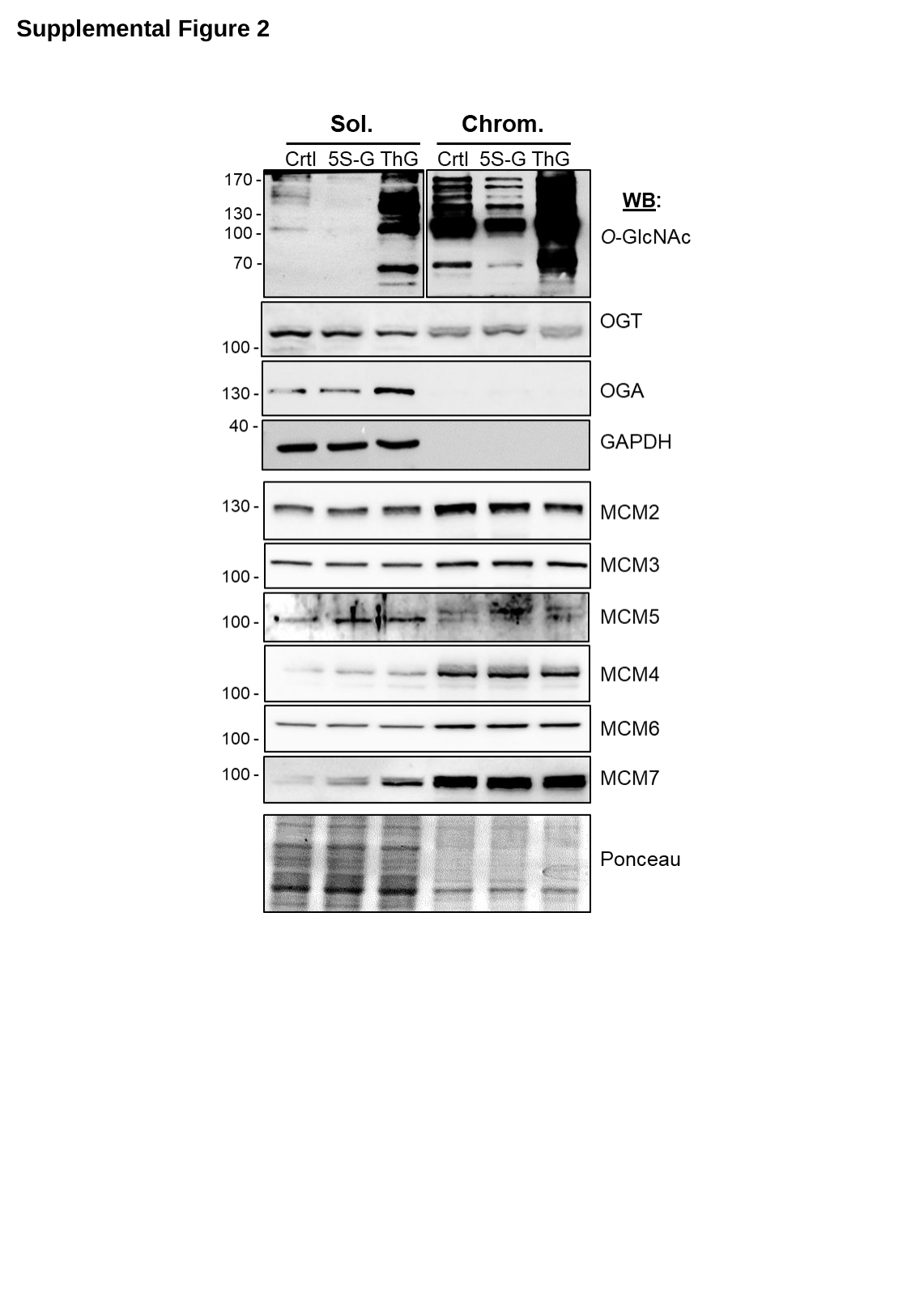

Supplemental Figure 2

## Slide 3
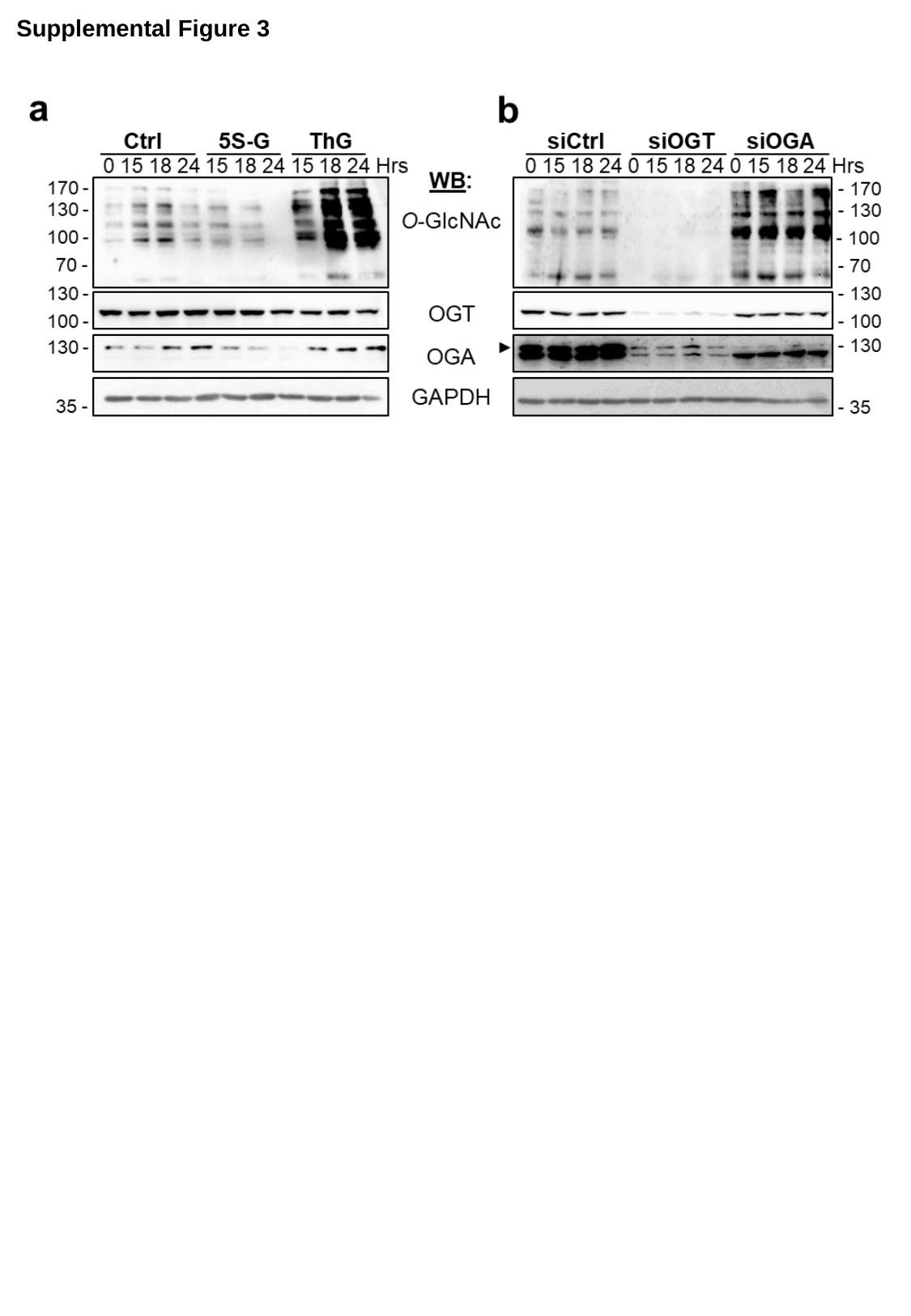

Supplemental Figure 3

Supplement: Supplementary file 1 — Supplementary Figure Captions: Suppl. Figure 1 O-GlcNAcylated MCM2-7 subunits are mainly found in the chromatin-enriched fraction in MDA-MB-231 cells. Serum-starved MDA-MB-231 cells were released into the cell cycle by serum addition for the indicated times. a Cell cycle distribution was determined by flow cytometry after DNA staining with PI. Percentage of cells in G0/G1, S and G2/M phases are indicated. b The nucleocytoplasmic soluble (Sol.) and chromatin-bound fractions (Chrom.) were analysed by Western blot for the indicated proteins. c O-GlcNAcylated proteins from both fractions were enriched on sWGA-agarose beads. Incubation with excess of GlcNAc (0.5 M) was used as negative control (C−). MCM proteins were detected by Western blot before (Input) and after enrichment on sWGA lectin (sWGA-bound). d Recombinant GST-tagged OGT (OGT–GST) and GST alone (GST) were immobilized onto Glutathione Sepharose beads. GST pull-down assay was performed using soluble (Sol.) and chromatin-bound (Chrom.) protein fractions from asynchronous MDA-MB-231 cells (Inp). Eluted MCM proteins were detected by Western blot and anti-GST antibodies were used to confirm the presence of the recombinant proteins after elution. Suppl. Figure 2 Efficiency of inhibition of OGT and OGA in MCF7 cells. Serum-starved cells were released in S phase by serum addition for 18 h in presence of DMSO (Ctrl), 5S-G (50 µM) or ThG (1 µM), before harvesting. Proteins from subcellular fractions (Sol. and Chrom.) were separated by SDS-PAGE and analysed by Western blot for the indicated proteins. Equal loading was confirmed by Ponceau staining of the nitrocellulose membrane. Suppl. Figure 3 Serum-starved MCF7 were released in S phase by serum addition for the indicated times. a DMSO (1/1000, Ctrl), 5S-G (50 µM) or ThG (1 µM) was added at the same time as serum. b cells were transfected with siRNA 24 h before serum starvation. Whole cell lysates were resolved by SDS-PAGE and analysed by Western blot for the indicated [file 18_2018_2874_MOESM1_ESM.pptx]
